# Sublethal effects of a vapour-active pyrethroid, transfluthrin, on *Aedes aegypti* and *Ae. albopictus* (Diptera: Culicidae) fecundity and oviposition behaviour

**DOI:** 10.1186/s13071-018-3065-4

**Published:** 2018-08-29

**Authors:** Christopher S. Bibbs, Daniel A. Hahn, Phillip E. Kaufman, Rui-de Xue

**Affiliations:** 1 Entomology and Nematology Department, University of Florida, 1881 Natural Area Drive, Gainesville, FL 32611 USA; 2 Anastasia Mosquito Control District of St. Johns County, 120 EOC Drive, St. Augustine, FL 32092 USA

**Keywords:** Repellent, Mosquito, Transfluthrin, Fecundity, Vector control

## Abstract

**Background:**

It is assumed that mosquitoes surviving exposure to spatial repellents when attempting to bite a host will not have significant adverse impacts on their downstream biology. Therefore, a critical knowledge gap is understanding the extent to which sublethal exposure to volatile pyrethroids may damage the performance of mosquitoes that survive exposure to vapour-active pyrethroids. To address this, laboratory-reared *Aedes aegypti* (L.) and *Ae. albopictus* (Skuse) were exposed to one of three sublethal concentrations of transfluthrin before being offered a blood-meal, after which their survival, fecundity, fertility, and egg-laying behaviour was assessed.

**Results:**

Both species expressed reduced skip-oviposition behaviour at all exposures. Both species also suffered a major reduction in viable eggs (50–75% reduction in viable eggs laid). A phenotype where eggs collapsed after laying was observed in *Ae. aegypti,* and this response increased with exposure concentrations. Dissected females of both species retained 50% or fewer of their eggs, with *Ae. albopictus* retaining a significant proportion of melanised oocytes following the highest exposure.

**Conclusions:**

Our findings suggest that volatile pyrethroids can reduce skip-oviposition, which may improve source reduction outcomes during integrated management. The additional fecundity reduction caused by sublethal exposures to volatile pyrethroids improves our confidence in recommending them for urban vector management. Furthermore, we suggest that volatile pyrethroids should be adapted into delivery methods compatible with mosquito abatement programs.

**Electronic supplementary material:**

The online version of this article (10.1186/s13071-018-3065-4) contains supplementary material, which is available to authorized users.

## Background

The emergence of tropical pathogens, particularly Zika virus and its link to congenital disabilities [1, 2] renewed emphasis on domestic *Aedes aegypti* (L.) and *Aedes albopictus* (Skuse) as urban disease vectors in the USA following local transmission of Zika virus in Florida [3]. These mosquito species disperse eggs across many natural and artificial containers holding small quantities of water that permeate urban landscapes [4]. These simplified aquatic ecosystems virtually eliminate larval predation pressure, resulting in source reduction being the only long-term pressure to reduce densities of larval habitat used by container-inhabiting mosquitoes. In some environments, targeting key oviposition sites with source reduction has been effective in reducing container-inhabiting mosquito density [5, 6]. However, *Ae. aegypti* and *Ae. albopictus* bet-hedge by using a skip-oviposition strategy [7, 8], whereby eggs are distributed across multiple developmental sites per clutch. Because of skip-oviposition, it is difficult to eliminate all of the numerous mosquito oviposition sites from a peridomestic landscape. Oviposition sites also are often cryptic and difficult to access, further limiting the effectiveness of source reduction and cultural control [4]. This places reliance on continuing to conduct chemical adulticiding, such as through ultra-low volume space sprays or outdoor residual treatments, which have variable rates of success [9, 10]. Consequently, personal repellents are an essential part of integrated vector management because it allows an extra layer of prevention when other treatments fail [3].

In the current market, topical repellents form the core of personal protection guidelines against mosquito vectors [3]. However, many people do not use topical repellents because they find them inconvenient to apply or unpleasant in smell or feel when applied. Spatial repellent devices, such as ambient emanators, mosquito coils, vaporiser mats, and liquid vaporisers are an alternative to topical repellents that are frequently chosen by consumers [9]. Spatial repellents allow the dissemination of active ingredients on small scales, often serving as personal protection devices [11–13]. When such devices emit volatile pyrethroids, these tools can both reduce mosquito contact with humans [14] and kill mosquitoes outright [12, 13]. This is due to the primary mode of action of pyrethroids, in which the active ingredient binds to the voltage-gated sodium channel (VGSC) as the primary mode of toxicity. As reviewed in other work [15, 16], volatile pyrethroids can bind to the VGSC after inhalation. This property of inhalation is a clear benefit of some active ingredients, such as metofluthrin or transfluthrin, that lead to various acute and sub-acute outcomes. Because of the combined benefits of repellency and mortality, volatile pyrethroids have been advocated as a tool for urban vector management [12, 13].

While volatile pyrethroids are repellents that can also cause mortality, mosquitoes have many opportunities to survive exposure. Mosquitoes may escape the area before acquiring a lethal dose, or strong air movement may disperse the vapours. This range of exposures potentially produces a suite of sublethal outcomes depending on what point the mosquito escapes exposure [15, 16]. Sublethal impairment of mosquito reproduction by spatial repellents is relevant for container-inhabiting mosquitoes because they live in close association with humans and have relatively short-range oviposition site use [8]. This allows spatial repellents to impact container-inhabiting mosquitoes when host-seeking, gravid, or ready for oviposition. Some mosquitoes survive and escape exposure to spatial repellents, while others show signs of toxicity after escaping, summarised in Achee et al. [15] and Bibbs & Kaufman [16]. The degree to which sublethal exposure to volatile pyrethroids in spatial repellents can negatively affect downstream fecundity or oviposition behaviours is unknown.

In related work, a wide array of experimental and commercial skin repellent compounds have been shown to deter *Ae. albopictus* from ovipositing in containers fitted with a repellent treated barrier or repellent contaminated water [17, 18]. Furthermore, Xue et al. [19] found that when oviposition sites were contaminated with DEET, gravid mosquitoes retained eggs for as much as three weeks after the exposure. Furthermore, the viability of these retained eggs decreased as more time elapsed before mosquitoes were able to deposit their eggs [19] successfully. Choi et al. [20] exposed gravid *Ae. aegypti* to a volatile pyrethroid, transfluthrin, and found that bacteria-baited oviposition cups were twice as attractive to treated mosquitoes and that treated mosquitoes had a less overall dispersion of eggs despite being offered multiple containers. Whether the observed changes in oviposition were due to disrupted skip-oviposition patterns is not discussed by that study. However, the growing observations of sublethal effects should be applied to the peridomestic ecology of *Ae. aegypti* and *Ae. albopictus* to quantify how spatial repellents affect mosquitoes surviving exposure. We quantified the effects of exposure to sublethal concentrations of transfluthrin vapours on *Ae. aegypti* and *Ae. albopictus* fecundity and oviposition behaviour to test the extent to which these chemicals damage mosquito reproductive performance. We predicted that exposing *Ae. aegypti* and *Ae. albopictus* to sublethal concentrations of volatile pyrethroid vapours would reduce mosquito fecundity in a subsequent oviposition attempt. Additionally, exposure to sublethal concentrations of volatile pyrethroid vapours was predicted to decrease the occurrence of skip-oviposition behaviour in both species.

## Methods

### Mosquito rearing

Pyrethroid susceptible, 1952 Orlando, FL strain *Aedes aegypti* and 1992 Gainesville, FL strain *Aedes albopictus* were acquired from the United States Department of Agriculture, Agricultural Research Service, Center for Medical, Agricultural, and Veterinary Entomology (USDA-ARS-CMAVE) in Gainesville, Florida, USA. Colonies of the susceptible strain were not exposed to insecticides before evaluation and were not supplemented with wild-type introductions. Mosquitoes were kept at 26 ± 1 °C, 85 ± 5% relative humidity (RH), with a 14:10 (L:D) photoperiod. Batches of 2000 eggs were placed in larval pans containing 2500 ml of reverse osmosis (RO) water. Hatched larvae were fed 1–3 g of liver and yeast mixture at a 3:2 ratio *ad libitum* in a 50 ml suspension. Maturing adult mosquitoes were kept in 30 × 30 × 30 cm flight cages. Flight cages also were supplied with 10% sucrose solution and RO water. Mosquitoes used at the start of experimentation were non-blood-fed, 5–7 day-old females that were given the opportunity to mate without blood-feeding.

### Bioassay design

Test cages were derived from single-use 473 ml clear polypropylene snap-lid cups. Container lids were modified to have a central 20 mm opening through which 20 female mosquitoes were aspirated into the container. The filter paper was cut into 5 mm widths and 40 mm lengths and pleated every 5 mm. Technical grade transfluthrin was serially diluted in acetone to create concentrations meeting LC_10_, LC_20_ and LC_30_ predicted dose responses using 24 h mortality data from Bibbs et al. [21]. The predicted concentrations from Bibbs et al. [21] were derived from acute toxicity dose responses after 2 h exposures, but the current concentrations were selected with the intent of avoiding acute mortality while still allowing signs of toxicity to be detected several days later. These concentrations in solution were 0.009% for LC_10_, 0.016% for LC_20_, and 0.026% for LC_30_ against *Ae. aegypti* and 0.012% for LC_10_, 0.020% for LC_20_, and 0.029% for LC_30_ against *Ae. albopictus*.

Paper strips were then treated with 40 μl of a transfluthrin solution. Treated strips were air dried for 6 min and then transferred into a mesh bag that was suspended within the test cage through the hole in the lid. The hole was sealed to prevent vapours from escaping during tests. Controls used paper strips treated with only acetone. Test cages were placed in an illuminated incubator maintained at 26 ± 1 °C, 85 ± 5% RH for a 2 h exposure period. The 2 h exposure was selected to adhere to the protocol of Bibbs et al. [21] and validate that the predicted concentrations abide the LC_10_, LC_20_ and LC_30_ acute toxicity outcomes.

Following the 2 h transfluthrin exposure, mosquitoes were removed into a clean container of the same design. Upon transfer to clean containment, both treatment and unexposed control cohorts were offered 100 ml of non-citrated bovine blood in an unsalted sausage casing after warming to 36 ± 0.5 °C using a hot water bath. Freshly warmed blood meals were supplied for 2 h per cohort, with blood replaced each hour. Post-blood-meal, mosquitoes lacking a fully engorged, red abdomen, as compared to control mosquitoes, were removed from the assay. The remaining mosquitoes were allowed 72 h post-blood meal to become gravid while held in the rearing conditions. Gravid mosquitoes for each cohort were confirmed with visual inspection of the abdominal membrane and transferred individually to polypropylene flight cages measuring 30 × 30 × 30 cm for oviposition (Bugdorm I # DP1000, MegaView Science Co. Ltd., Taichung, Taiwan). Groups of five flight cages, each with one mosquito per cage, were used for LC_10_, LC_20_ and LC_30_ treatments, and another group of five cages were used for control, totalling 20 cages per replicate. Each flight cage contained a source of 10% sucrose solution and six black, 118 ml oviposition cups (P400BLK, Dart Container Corporation, Mason, MI, USA). Each cup was filled with 50 ml of RO water and a 5 × 8 cm strip of oviposition paper (#6,512,981,311, Anchor Paper Co., Saint Paul, MN, USA). Gravid mosquitoes were allowed 72 h to oviposit across the array of cups. The parameters recorded from the oviposition bioassay included how many eggs were deposited, how many cups contained eggs, and the living or deceased status of the treated female mosquito. The flight-oviposition cages were randomly assigned a 0°, 90°, 180° or 270° orientation within an insectary at the beginning of the study to reduce the light effects and orientation biases.

**Fig. 1:**
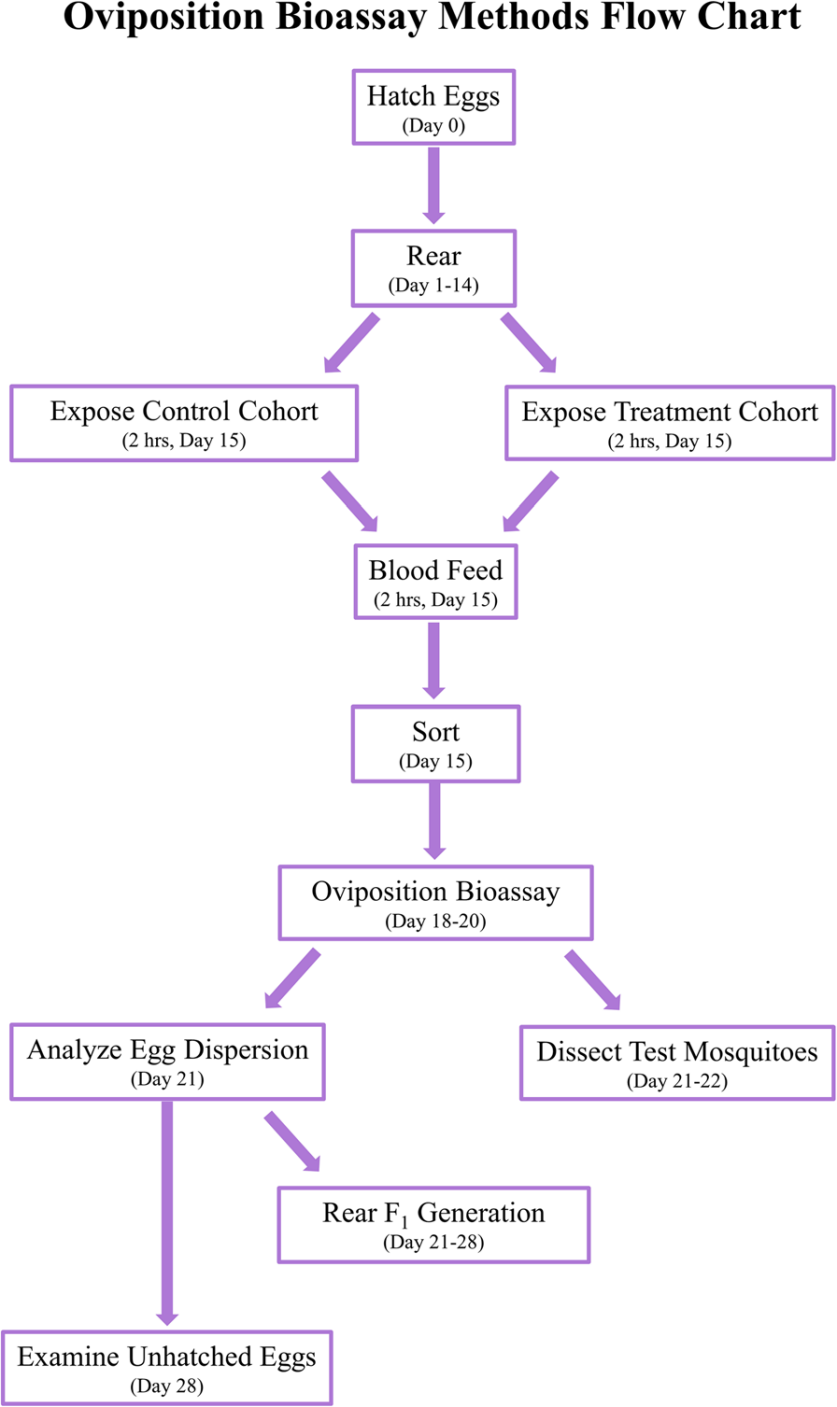
Flow chart: stages of the experiment with time estimates showing the step durations

Once oviposition bioassays were concluded, adult mosquitoes were immediately killed and dissected in 70% EtOH under a light microscope to examine egg retention. Dispersion of eggs laid across cups and quantity of eggs for each cup were recorded upon removal of adults for dissection. To facilitate embyronation of the eggs, oviposition papers were stored in rearing conditions and loosely enveloped in wax paper for a 24 h damp drying period. Eggs were inspected for any deformities following damp drying and then submerged in 118 ml cups filled with 50 ml of RO water. Larvae were reared out post-hatch with larval food provided *ad libitum* until all larvae pupated or perished. The number of larvae that hatched and subsequent natal mortality was recorded daily over the rearing period. Unhatched eggs were examined for deformities again once larval rearing from the selected paper was concluded. Eggs were denoted as non-viable if at either examination the chorion was collapsed. The progression required to complete a repetition of this bioassay appears in Fig. 1. This design was replicated across six different cohorts of mosquitoes for each species.

### Data analysis

Kruskal-Wallis analysis of variance and *post-hoc* Steel-Dwass tests were performed on averages of eggs collected, number of cups used by females, number of larvae successfully hatched and reared, number of eggs that failed to embryonate, and the number of eggs retained in dissected adult females after the bioassay to compare effects across treatments. Delayed toxicity of adult females for each treatment, whereby *Ae. aegypti* that were exposed to transfluthrin before blood-feeding were discovered in a prone position rigidly immobilised during the oviposition bioassay from 24 h to 6 d after transfluthrin exposure was compared to the controls using a Chi-square analysis. For *Ae. albopictus*, a melanisation effect observed in retained eggs also was analysed for each treatment using a Chi-square analysis. The severity of oocyte melanization in each female was qualitatively placed into one of five groups (0% of oocytes melanized; 1–25% of oocytes melanized; 26–50% of oocytes melanized; 51–75% of oocytes melanized; and 76–100% of visible oocytes melanized) and proportions of each group were compared among treatments. Statistical procedures were repeated for all concentrations and species in JMP 13.1.0 (SAS Institute, Inc., Cary, NC, USA).

## Results

### *Aedes aegypti*

**Fig. 2:**
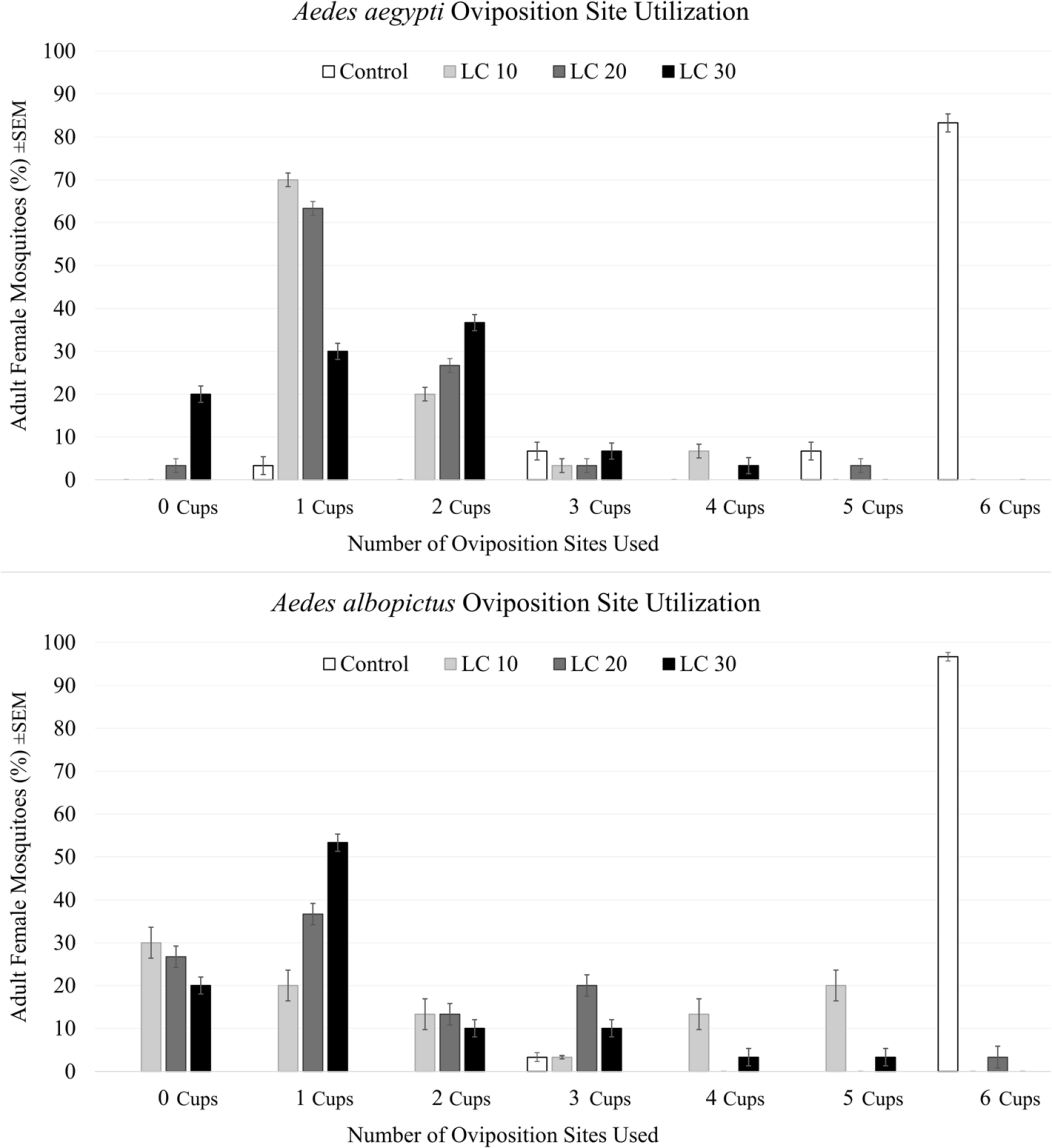
Cluster graphs representing the mean percentage of adult *Aedes aegypti* (L.) (*n* = 30) and *Ae. albopictus* (Skuse) (*n* = 30) ovipositing in 0, 1, 2, 3, 4, 5 or 6 cups following exposure to three sublethal concentrations of transfluthrin (LC_10_, LC_20_, LC_30_). Figures shown with a standard error of the mean as I-bars

**Fig. 3:**
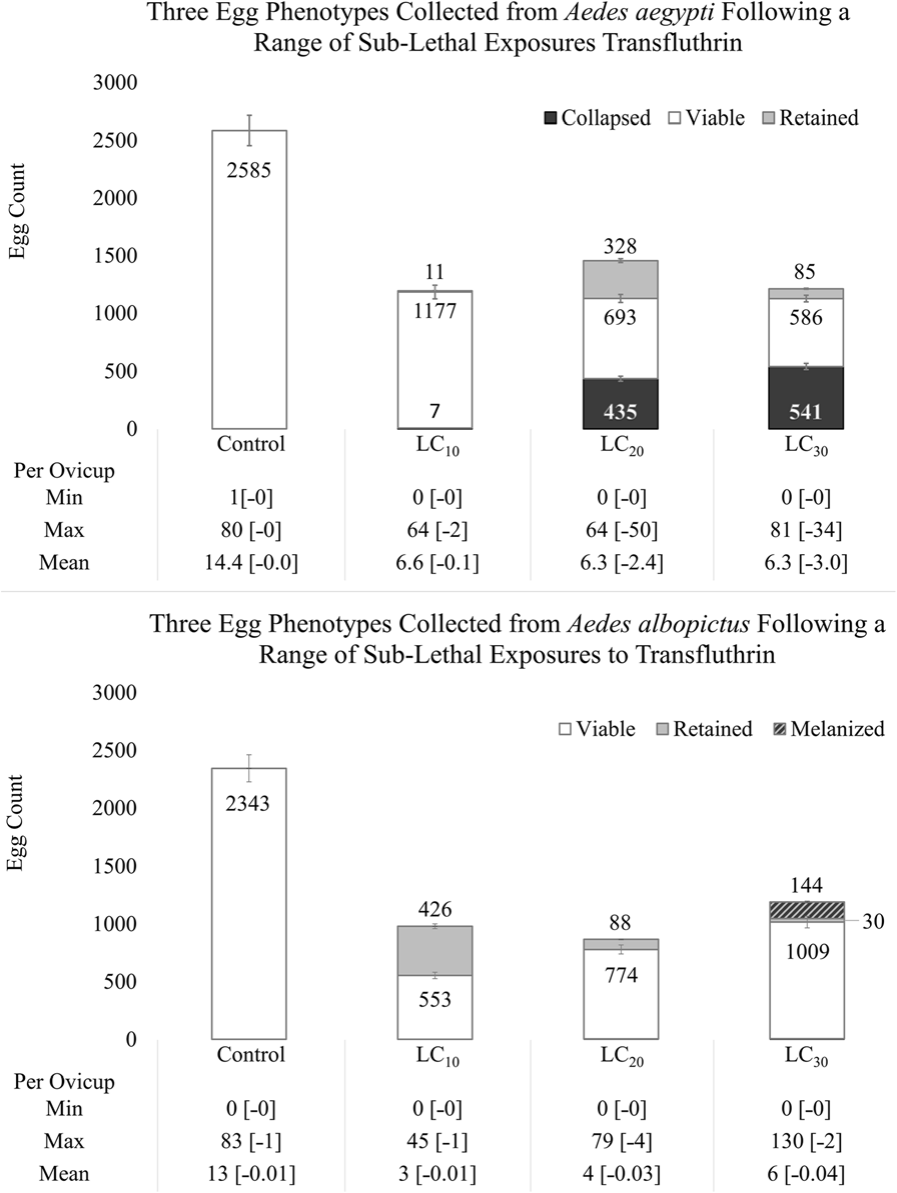
The LC_10_, LC_20_, and LC_30_ of transfluthrin were tested on *Aedes aegypti* (L.) (*n* = 30) and *Ae. albopictus* (Skuse) (*n* = 30). Compound bar graphs represent the counts of collapsed eggs (*Ae. aegypti*), viable eggs, eggs retained in the parent female, and melanisation of retained eggs (*Ae. albopictus*) following oviposition in a six-cup arena. The sums of: Collapsed or Melanized + Viable + Retained = total egg production, Collapsed + Viable = total eggs oviposited, Viable + Retained = yield that could recruit to the next generation. Figures are shown with 95% confidence as I-bars. Tables beneath graph detail range and mean counts per ovicup in the bioassay as ‘Viable [˗Collapsed]’

Exposure of female *Ae. aegypti* to sublethal concentrations of transfluthrin reduced the dispersion of eggs across the oviposition arena (Fig. 2, *χ*^2^_(3)_ = 66.2, *P* < 0.0001). All transfluthrin exposure groups oviposited in fewer cups than the unexposed controls, but there were no differences in egg dispersion between our volatile-pyrethroid exposure groups (mean ranks from Kruskal-Wallis tests were 103.2 for controls, 46.6 for LC_10_, 46.3 for LC_20_, and 45.9 for LC_30_). There was also a significant reduction in the total number of eggs oviposited by *Ae. aegypti* females following sublethal transfluthrin exposure compared to unexposed controls (Fig. 3, *χ*^2^_(3)_ = 187.6, *P* < 0.0001, *n* = 30), but there were no differences in total numbers of eggs oviposited among our volatile-pyrethroid exposure groups (mean ranks from Kruskal-Wallis tests were 524.5 for controls, 308.4 for LC_10_, 306.1 for LC_20_, and 303.0 for LC_30_).

**Fig. 4:**
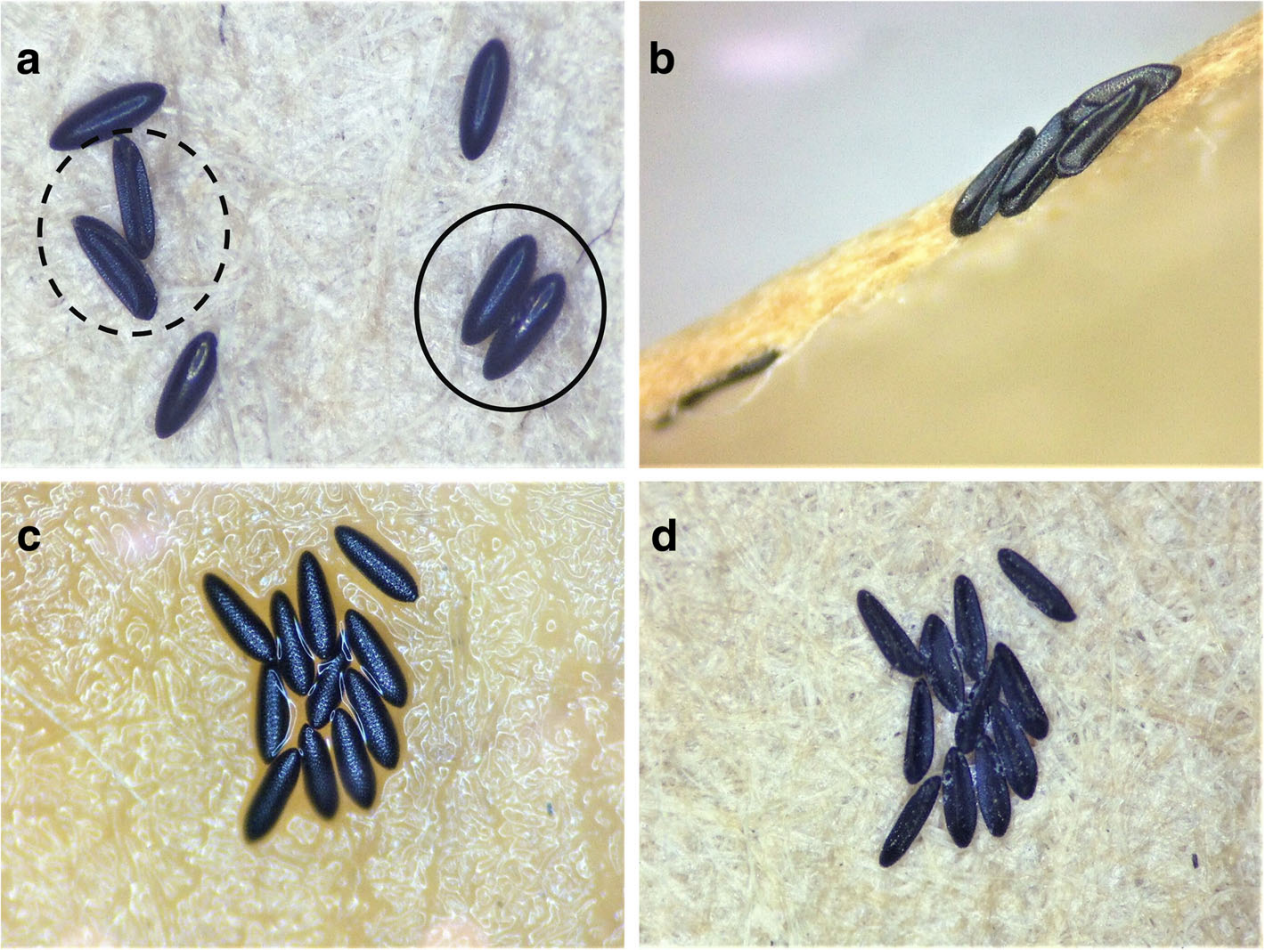
Egg collapse: *Aedes aegypti* (L.) eggs following exposure of the parent female mosquito to sublethal concentrations of transfluthrin. **a** Seed germination paper removed from an LC_10_ treatment oviposition bioassay cup, following a 24 h damp dry, and a full drying period. Solid line encircles embryonated, viable eggs. Dashed line encircles non-viable eggs with a collapsed chorion. **b** Cluster of collapsed eggs from an LC_20_ treatment oviposition bioassay, following a 24 h damp dry period, but not fully dried. **c** A cluster of eggs upon immediate removal from an LC_30_ treatment oviposition bioassay. **d** The same cluster of eggs, collapsed, following a 24 h damp dry and full drying period

Female *Ae. aegypti* exposed to transfluthrin also experienced reduced egg viability. This was evident because many eggs were collapsed and few hatched in our sublethal exposure groups (Fig. 4b, d). Collapsed eggs tended to occur in clusters but were sometimes mixed with viable eggs (Fig. 4a). In ~30% of samples, the entire collection of eggs on an oviposition paper were collapsed by the end of the larval hatching period. The LC_10_ exposure group did not significantly differ in the number of collapsed eggs from the controls. However, the LC_20_ and LC_30_ exposures had significantly higher numbers of collapsed eggs than those observed in either the LC_10_ or control treatments (Fig. 3, *χ*^2^_(3)_ = 76.0, *P* < 0.0001, *n* = 30; mean ranks from Kruskal-Wallis tests were 320.0 for controls, 393.6 for LC_20_, and 401.1 for LC_30_).

Eggs that were not collapsed were considered potentially viable, therefore collapsed eggs were removed from the total, and only the remaining eggs were hatched out. Of these, the larval hatch was not significantly different across treatments and controls, but a weak visual trend indicated a negative correlation of hatch rate with exposures. In rearing the viable eggs, there was no difference in the survivorship to adulthood of hatched larvae between the control, LC_10_, and LC_20_ treatments. There was a weak trend suggesting that survivorship decreased 2% in larvae hatching from the eggs laid by LC_30_-exposed female mosquitoes (*χ*^2^_(3)_ = 11.0, *P* < 0.0116), with a mean rank of 373.6 for control, 368.5 for LC_10_, 351.8 for LC_20_, and 348.1 for LC_30_. There were no effects on pupation, adult emergence, or subsequent blood-feeding and oviposition in the F_1_ generation.

There was significantly delayed toxicity at the higher exposure levels where *Ae. aegypti* that were exposed to transfluthrin before blood-feeding were discovered dead six days after exposure when concluding oviposition bioassays. The LC_20_ exposure generated 36.7% delayed toxicity in adult females during the oviposition bioassay, and the LC_30_ exposure generated 60.0% delayed toxicity (*χ*^2^_(3)_ = 52.9, *P* < 0.0001). No delayed toxicity was observed in LC_10_ or the controls. Even though they may have died soon after, all females that died during the oviposition bioassay survived long enough to lay eggs in the provided arena. Both living and dead mosquitoes were dissected after the oviposition bioassay to determine whether they retained any matured eggs in their reproductive tracts or whether all matured eggs were laid. During dissections, control mosquitoes did not retain any mature eggs, while the egg retention in the LC_10_, LC_20,_ and LC_30_ cohorts ranged from 0–3, 0–28, and 0–27 eggs per female, respectively. Females in the LC_20_ group retained more mature eggs than controls (Fig. 3, *χ*^2^_(3)_ = 61.5, *P* < 0.0001, mean rank of 39.5 for control, 48.4 for LC_10_, 95.3 for LC_20_, and 58.8 for LC_30_). Although there was an observed trend towards egg retention in the LC_30_ group, this trend was not statistically detectably different from the control. Overall, when we combine each of the facets of reduced reproductive performance that we quantified in *Ae. aegypti,* mosquitoes exposed to the LC_30_ dose of transfluthrin vapours had ~70% reduction in viable eggs.

### *Aedes albopictus*

*Aedes albopictus* also oviposited across significantly fewer sites than control mosquitoes when exposed to any of the sublethal concentrations of transfluthrin (Fig. 2, *χ*^2^_(3)_ = 67.6, *P* < 0.0001, *n* = 30; Kruskal-Wallis mean ranks of 104.4 for control, 50.5 for LC_10_, 44.3 for LC_20_, and 42.8 for LC_30_). As above, there was a significant reduction in the total number of eggs deposited by mosquitoes exposed to any of the transfluthrin concentrations compared to controls (Fig. 3, *χ*^2^_(3)_ = 242.7, *P* < 0.0001, *n* = 30; mean ranks of 550.5 for control, 315.0 for LC_10_, 286.1 for LC_20_, and 290.4 for LC_30_).

**Fig. 5:**
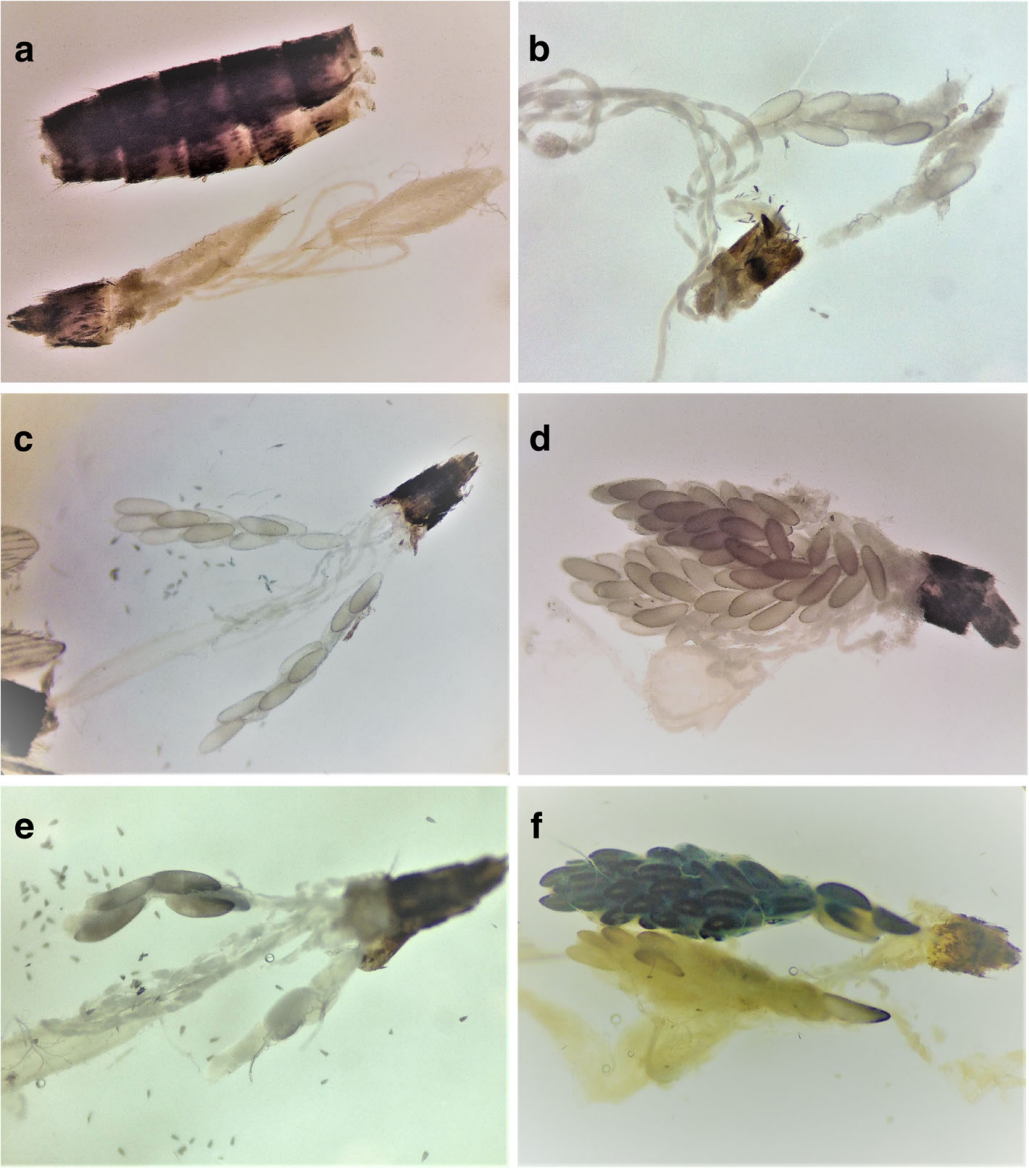
Egg melanization: dissected female *Aedes albopictus* (Skuse) reproductive tracts after exposure to sublethal concentrations of transfluthrin and upon conclusion of oviposition bioassays seven days post-exposure. **a** Control mosquito showing cleared oviducts and no late-stage development oocytes. **b** A reproductive tract following LC_10_ treatment with 10 or fewer retained eggs. **c** A reproductive tract following LC_20_ treatment with 20 or fewer retained eggs. **d** A reproductive tract following LC_30_ treatment with extreme egg retention. **e** A reproductive tract following LC_30_ treatment showing partial melanisation of retained eggs. **f** A reproductive tract following LC_30_ treatment showing full melanisation and tissue decay.

In contrast to *Ae. aegypti*, less than 1% of *Ae. albopictus* eggs were collapsed across all treatments and controls. Larval hatch was 98–100% successful for the control and all transfluthrin treatments, and larval survival after hatching was not significantly different across transfluthrin treatments and the control. Unlike *Ae. aegypti*, there was no delayed toxicity observed in adult *Ae. albopictus* as a result of exposure to transfluthrin vapours. When female mosquitoes were dissected to determine whether all matured eggs were laid, we found that *Ae. albopictus* controls did not retain any mature eggs, but the LC_10_ group collectively retained more mature eggs than either the control or higher-concentration transfluthrin treatments (Fig. 3, Fig. 5, *χ*^2^_(3)_ = 54.8, *P* < 0.0001, *n* = 30; mean ranks of 32.0 for control, 90.3 for LC_10_, 50.2 for LC_20_, and 69.5 for LC_30_). Females in the LC_30_ treatment retained significantly more eggs than the control (*Z* = 5.5, *P* < 0.0001) and LC_10_ (*Z* = -3.1, *P* < 0.0109) groups, but were not statistically different from the LC_20_ group.

During dissections, we found that in the LC_30_ treatment group 70% of the females contained a proportion of oocytes retained in the reproductive tract that were melanized (Fig. 5e, f), but neither the control nor other sublethal exposure groups had melanized oocytes (Fig. 5a-d, *χ*^2^_(12)_ = 74.6, *P* < 0.0001). The ratio of melanised eggs with respect to retained and oviposited eggs are displayed in Fig. 3. If melanised eggs were not viable, the combined effects that reduce reproductive performance in *Ae. albopictus* represent ~65% total reduction of viable eggs after exposure to the LC_30_ of transfluthrin vapours.

## Discussion

Exposure to sublethal concentrations of transfluthrin vapours reduced female reproduction, including fecundity, fertility, and the dispersion of eggs across potential oviposition sites in both *Aedes* species. The breadth of impacts to reproductive performance add to the spectrum of outcomes against mosquitoes when using spatial repellents. Skip-oviposition, in particular, is a critical behaviour to interrupt because untreated *Ae. aegypti* and *Ae. albopictus* consistently spread eggs to 4–6 containers per gonotrophic cycle, as seen in our control mosquitoes [22–25]. However, skip-oviposition is susceptible to external pressures, it wanes if there are limited oviposition sites available [25], during seasonal changes [23], and in certain geographical localities [26]. Source reduction ideally should eliminate skip-oviposition behaviour by removing options for oviposition [25]. In practice, it has become evident that container-inhabiting mosquitoes find oviposition sites despite source reduction efforts, thereby confounding the sustainability of source reduction as a mosquito abatement strategy [6]. Volatile pyrethroids may provide an additional external pressure needed to manipulate skip-oviposition behaviour in *Ae. aegypti* and *Ae. albopictus* to facilitate source reduction impacts within an integrated approach.

Our results represent both a dramatic reduction of viable eggs and a favourable reduction of skip-oviposition behaviour by both *Ae. aegypti* and *Ae. albopictus* upon exposure to transfluthrin. The changes in oviposition behaviour in our work may be a result of behavioural modification by neuronal interference since transfluthrin binds to the VGSC. A complementary effect was discussed by Choi et al. [20] where *Ae. aegypti* displayed increased attraction to oviposition sites after sublethal exposure to transfluthrin. When paired with the reduced reproductive performance observed in the present study, spatial repellents appear to stimulate container-inhabiting mosquitoes to oviposit in nearby containers urgently. Therefore, volatile pyrethroids can synergise with source reduction programs by reducing the labour necessary to target key oviposition sites [5]. Additionally, there are other circumstances where mosquitoes may get unintentional sublethal exposures. It has suggested that the time it takes for the vapours to penetrate into surroundings can lead to reduced exposure of target mosquitoes [26, 27]. Our findings also support that mosquitoes that may escape spatial repellents can still incur various side-effects. A mosquito that approaches hosts protected by spatial repellents on multiple occasions may even experience several repeated sublethal exposures through its lifetime.

However, the current delivery methods for vapour-active pyrethroids as spatial repellents are restricted to managing small areas. With current delivery methods protecting large areas would be as labour intensive and costly as source reduction [5, 6]. Given the ecology of domestic mosquitoes and the growing spectrum of benefits that volatile pyrethroids have against container-inhabiting mosquitoes, volatile pyrethroids should be developed into tools that are more capable of addressing the needs of mosquito abatement programs. Recent field studies support the idea that vapour-active pyrethroids can be deployed in ultra-low volume sprays to suppress domestic mosquitoes [9, 10]. Vapour-active pyrethroids also may be compatible with other delivery formats that are useful for integrated vector management.

Although our results are proof of concept that spatial repellents can harm mosquito reproduction six days after exposure, many details are not well understood and will require further study. When breaking apart the fertility measurements, *Ae. albopictus* had an unusual pattern whereby all of the treatments had lower egg viability than the control, but there was an increasing pattern of egg viability with higher transfluthrin concentration. A possible explanation is hormesis [28], a well-documented occurrence in which an organism (e.g. insects) experiences sublethal exposure to a stressor (e.g. toxicant or xenobiotic) and correspondingly displays increased fitness (e.g. egg production) at low doses while being inhibited at high doses. [29, 30]. Hormesis is an effect observed in both field and laboratory test groups, and current evidence asserts that pesticide hormesis does not significantly differ in magnitude in the laboratory *versus* field colonies for *Ae. aegypti* [30]. Unfortunately, our range of concentrations was not broad enough to observe a drop in the effect after a peak, so we cannot confirm what caused the pattern in viability or what it means for the animal. The effect was minimal for overall reproduction, and the net effect still resulted in less oviposition compared to the control group. In contrast, the oocyte melanisation observed in *Ae. albopictus* could be explained as an immune-system cost of surviving exposure. Cellular responses to stress, such as upregulation of detoxification enzymes or immune responses, have been shown to trigger melanisation in the ovary and follicular apoptosis in *Anopheles gambiae* Giles [31]. In one *Ae. albopictus* female with 100% of the visible oocytes melanised, bacterial decomposition was visible in one of the ovaries of the otherwise surviving female mosquito. Stress responses could explain the observed melanisation and tissue decay, reinforcing that sublethal exposures to volatile pyrethroids could have substantial implications for mosquito populations.

## Conclusions

There are many potential directions for continuing work on sublethal effects caused by volatile pyrethroids. Although we monitored for mortality and reproductive performance for one week after exposure and blood-feeding, there may be long-term effects of sublethal exposures beyond the first gonotrophic cycle that should be examined. Exposure concentrations also can be controlled by time and have been recognised as a relevant limiter for volatile pyrethroids when the vapours must travel and penetrate into areas of interest [26, 27]. To make the findings of our proof of concept study more relevant to field conditions, a valuable next step would be to test short exposure durations. Particularly with respect to the fact mosquitoes may only briefly encounter the toxicant when approaching hosts. Regardless, this study demonstrated that even despite hormetic gains in one facet of reproduction, *Ae. aegypti* and *Ae. albopictus* experienced reduced overall egg yield, viability, and skip-oviposition behaviour following exposure to transfluthrin at a low concentration.

## Additional file

Additional file 1: **Table S1.** Raw data for Figs. 2–3. (XLSX 186 kb)

## Abbreviations

LC: Lethal concentration
USDA-ARS-CMAVE: United States Department of Agriculture, Agricultural Research Service, Center for Medical, Agricultural and Veterinary Entomology
